# The efficacy of a porcine collagen matrix in keratinized tissue augmentation: a 5-year follow-up study

**DOI:** 10.1186/s40729-017-0113-3

**Published:** 2018-01-10

**Authors:** C. Maiorana, L. Pivetti, F. Signorino, G. B. Grossi, A. S. Herford, M. Beretta

**Affiliations:** 1 Oral Surgery, Center for Edentulism and Jaw Atrophies, Maxillofacial Surgery and Dentistry Unit, Fondazione IRCCS Cà Granda—Ospedale Maggiore Policlinico, University of Milan, Milan, Italy; 2 Center for Edentulism and Jaw Atrophies, Maxillofacial Surgery and Dentistry Unit, Fondazione IRCCS Cà Granda—Ospedale Maggiore Policlinico, University of Milan, Via della Commenda 10, 20122 Milan, Italy; 3 Department of Oral Surgery, School of Dentistry, University of Milan, Milan, Italy; 4 Department of Oral & Maxillofacial Surgery, Loma Linda University, Loma Linda, CA USA

**Keywords:** Collagen matrix, Keratinized tissue, Mucosal grafts, Xenogeneic graft

## Abstract

**Background:**

When keratinized tissue width around dental implants is poorly represented, the clinician could resort to autogenous soft tissue grafting. Autogenous soft tissue grafting procedures are usually associated with a certain degree of morbidity. Collagen matrices could be used as an alternative to reduce morbidity and intra-operatory times. The aim of this study was to assess the efficacy of a xenogeneic collagen matrix as a substitute for soft tissue grafting around dental implants.

**Methods:**

Fifteen consecutive patients underwent a vestibuloplasty and keratinized tissue reconstruction around dental implants, both in the mandible and the maxilla, with a porcine collagen matrix. The so obtained keratinized tissues were measured and evaluated after 6 months and 1, 4, and 5 years.

**Results:**

The average gain of keratinized tissue was 5.7 mm. After 6 months, it was observed a resorption of 37%, after 1 year 48%, and after 5 years 59%. The mean gain of keratinized tissue after 5 years was 2.4 mm. Hemostatic effect and post-operative pain were evaluated too. All subjects referred minimal pain with no bleeding. No adverse reaction nor infection was noted.

**Conclusions:**

The present study showed the efficacy of a porcine collagen matrix in keratinized tissue augmentation. The possibility to use a soft tissue substitute is a great achievement as morbidity decreases and bigger areas can be treated in a single surgery.

## Background

A variety of factors can lead to teeth loss. From periodontal disease to trauma, the bone remodeling that always follows this event can complicate the subsequent prosthetical rehabilitation [1]. Both removable and implant-fixed restorations require both an adequate quantity of bone and sorrounding soft tissue. Even in severe atrophies of the jaw, nowadays, many bone augmentation techniques are applicable, all with an acceptable long-term stability [2]. Unfortunately, these techniques, especially in major reconstructions, lead to a deficient quantity of soft tissue, above all keratinized mucosa. Whether or not the presence of keratinized tissue around dental implants is necessary, it has been controversial for many years. The lack of evidence in literature regarding implant survival rate in absence of keratinized tissue cannot lead to any conclusion [3]. Nonetheless, the presence of this kind of tissue is desirable for a number of reasons, as shown by many authors. A retrospective study, based on 339 implants, showed that a lack of keratinized tissue around dental implants, especially in the posterior region, led to higher plaque accumulation and mucositis [4]. Thin or narrow (< 1 mm) peri-implant keratinized mucosa was shown to have a higher association with mucosal recessions [5]. At the same time, another retrospective evaluation of 250 implants after 5 to 10 years of functional loading demonstrated a negative correlation between the presence of keratinized tissue and mucosal recessions [6]. Consequently, it is safe to say that sufficient peri-implant keratinized tissue prevents peri-implant plaque accumulation and buccal soft tissue recessions, accordingly reducing the risk of mucositis and peri-implantitis [7, 8]. A surgical technique based on an apically positioned flap (APF) for vestibuloplasty associated with grafting materials (whether autologous or not) is considered the gold standard for soft tissue augmentation [9]. While a number of autologous grafts have been studied in the past, free gingival grafts from the palatal region are considered the most reliable and effective, in spite of the high morbidity of this type of surgery and extremely poor esthetics [10, 11]. In the past few years, collagen matrices (CM) have been studied as a valid substitute for free gingival grafts, in particular the porcine CM Mucograft (Geistlich Biomaterials GmbH, Baden-Baden, Germany). So far, a number of studies have demonstrated that the Mucograft is reliable and comparable with free gingival graft for what concerns achievements in keratinized tissue augmentation around both teeth or dental implants [12–15]. Additional advantages are a lower patient morbidity due to the absence of a donor site and the high esthetic value, matching texture and color of the adjacent mucosa [11, 15–17]. Concerns about long-term stability of this kind of procedure are more than reasonable, as the majority of papers in literature have short-term follow-ups (< 1 year), while just a few extend over this period. The aim of the present research is to evaluate the efficacy of the Mucograft in a standard APF procedure over 5 years of follow-up.

## Methods

The study was designed as a multicentered (Milan University—School of Dentistry/Loma Linda University—School of Dentistry) prospective observational (non-controlled) clinical study according to the STROBE criteria. The participants of the study presented areas of deficient attached and unattached mucosa precluding the construction of effective functioning prosthesis. The study included a total of 15 patients, both female and male, who were candidates for mucosal soft tissue augmentation by means of a xenogeneic CM (Geistlich Mucograft®, Geistlich Pharma AG, Wolhusen, CH). Patients included had to be at least 18 years old, both systemically and periodontally healthy with good oral hygiene; patients who were heavy smokers or bore systemic diseases that could influence bone turnover/wound healing were excluded. The purpose of the surgery was to improve the quantity of attached and unattached mucosa in order to facilitate the final prosthetic rehabilitation. The entire study was reviewed and approved by the Ethics Committee of the IRCCS Ospedale Maggiore Policlinico di Milano, Fondazione Ca’ Granda. Written consent was obtained at the recruitment visit from all the participants.

### The material

The xenogeneic CM (Mucograft®) is a class III medical device according to the Medical Device Directive 93/42 (EEC definitions: 1.1, long-term implant; 1.2, implantable; 8, resorbable; and 17, porcine origin). Its structure consists of two functional layers: a cell occlusive layer consisting of collagen fibers in a compact arrangement and a thick porous layer. This porous layer provides a space that favors the formation of a blood clot and the ingrowth of tissue from adjacent sites. This xenogenic graft has been cleared by the EU and US Food and Drug Administration for regenerative therapy involving teeth and implants.

### The surgical procedure

The surgical procedure, as already described in a previous study, consisted in a standard apically positioned flap with subsequent apposition of the CM, performed under local anesthesia [17]. At first, a midcrestal incision was performed in the residual keratinized band and a split thickness flap was raised. Any muscle fibers and fibrous banding attached in the area were dissected from the periosteum and were reduced toward the depth of the vestibule. In addition, submucosal fatty tissue was also dissected from the periosteum over the bone in the area. The lateral portion of the mucosal flap was sutured to the periosteum in the depth of vestibule. The denuded area of the wound in the vestibule was then covered by the CM. The CM was sutured to the surrounding mucosa anteriorly, posteriorly, and in the vestibular direction. The suturing was by 5–0 nylon. Approximately half of these sutures were allowed to remain in place for a period of 4 to 6 weeks to determine the outline of the original periphery of the incision and “graft” post-operatively. This was important in determining the area of actual re-epithelization of the defect and in determining and quantifying any shrinkage by scarring in the grafted area. Time was taken from the first incision to the last suture to record and compare the surgery length. A previously prepared acrylic splint was placed over the vestibuloplasty site at the time of surgery. This splint remained in place for 10 days at which time it was removed. The patient had to irrigate the area with 0.9% NaCl solution for those 10 days and rinse with 0.2% chlorhexidine.

### Specific endpoints and clinical follow-up

**Fig. 1:**
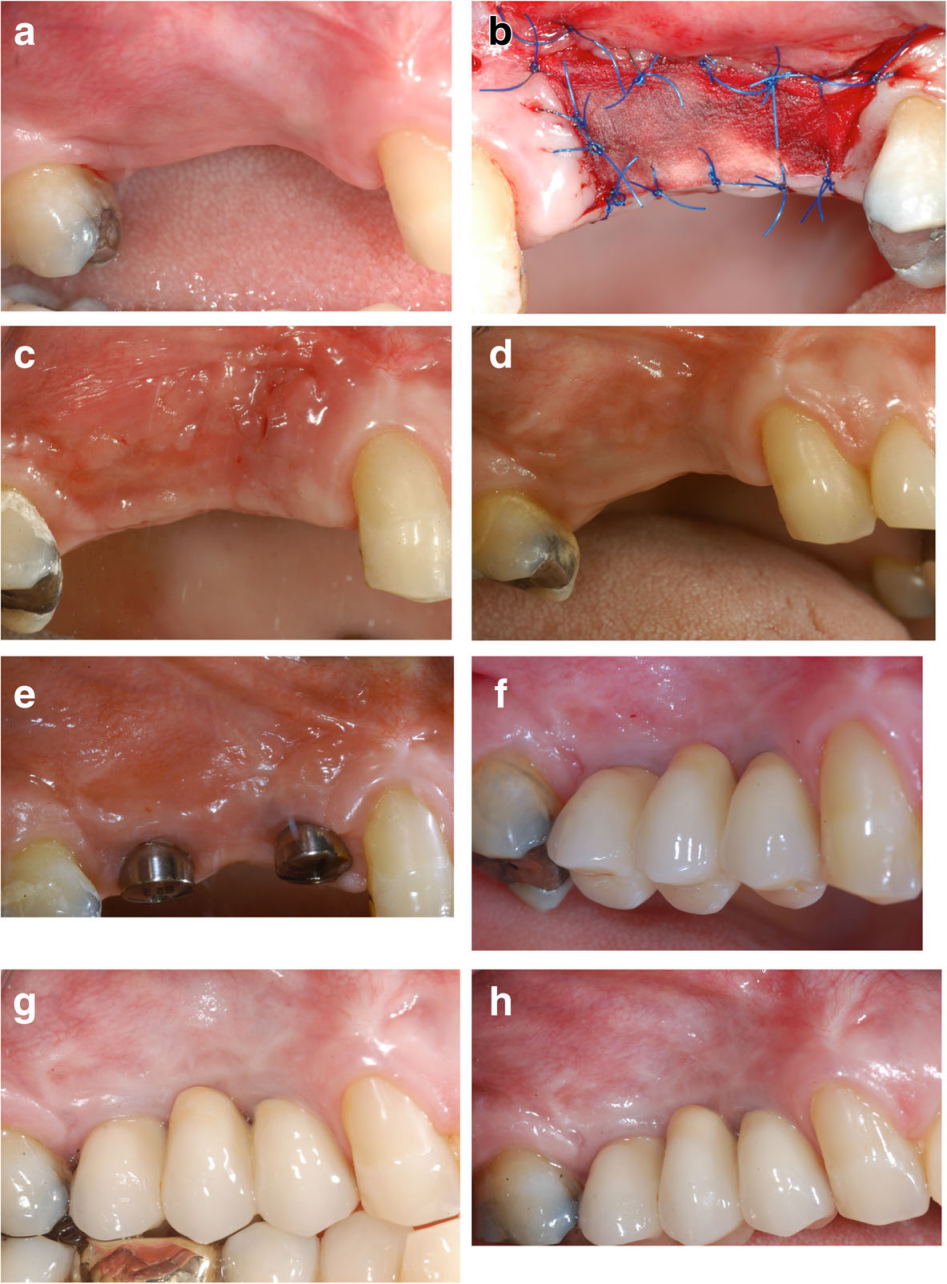
**a** Pre-op. **b** Post-op. **c** Two weeks after surgery. **d** Four weeks after surgery. **e** Six months after surgery. **f** One year after surgery. **g** Four years after surgery. **h** Five years after surgery

The primary endpoints were to evaluate the shrinkage degree of the width of keratinized mucosa and length of the re-epithelization process. The secondary endpoints assessed clinical evaluation of the grafted area, post-operative hemostatic effect, pain level, and length of surgery. Follow-up control visits were scheduled at 3 days after surgery and then 10 days, 2 weeks, 3 weeks, 1 month, 2 months, 6 months, 1 year, and 4 and 5 years as showed by the clinical case reported in Fig. 1. At each examination time point, the width of keratinized tissue (recorded from the crestal to the apical sutures; 3 to 5 measurement from mesial to distal) and vestibular depth were recorded by means of a 15-mm North Carolina periodontal probe. Once the implants were placed, measurement was taken from the free gingival margin, at the prosthetical crown’s zenith. Re-epithelization was evaluated clinically after 4 weeks on a scale from excellent (100% of the grafted area) to poor (< 40%). The degree of healing and maturation of tissues were observed and compared to the physiological healing time. Digital pictures were taken at each examination for comparison with the adjacent soft tissue. Hemostatic effect and pain level, evaluated using the Mankoski Pain Scale (from 0 to 10 where 0 is “No Pain” and 10 is “Pain makes you pass out”), were recorded until the 10th day (or whenever an increase of discomfort/bleeding was reported by the patient); examination time point in which the protective acrylic splint was removed [18]. Sutures were left in place for 4 weeks in order to facilitate the recording during healing. In all cases, dental implants were placed 2 months after the CM grafting elevating a full-thickness flap accessing the crestal bone. No differences between the original and the newly formed keratinized tissue were clearly appreciable so the flap was designed without any particular modifications. No bone grafting procedures were needed. Healing abutments were placed following a golden standard protocol, consisting in 4 months healing for the maxillary implants and 3 months for the mandibular ones.

### Statistical significance

Since a split-mouth design was not feasible and the defects being corrected by the mucosa particularly in the vestibular portion of the study are not usually symmetrical or bilateral, the use of paired subjects was not a reliable format. All the data were analyzed with IBM’s SPSS Statistics using ANOVA Repeated Measurements statistical method. Mean values for keratinized mucosal width and probing depth were recorded in millimeters at each examination time point and subsequently expressed in a percentage. First measurement for keratinized tissue, recorded immediately after surgery, was considered as 100%. Less than 20% of contraction was considered as excellent value, while good when less than 50%, fair to poor when higher than 50%, and unsatisfactory when the graft was lost.

## Results

**Fig. 2:**
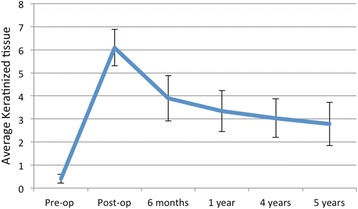
Average keratinized tissue (in mm)

**Fig. 3:**
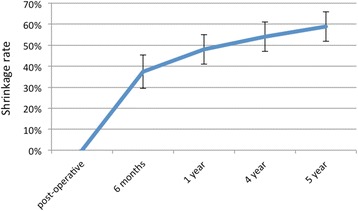
Shrinkage rate during time

**Fig. 4:**
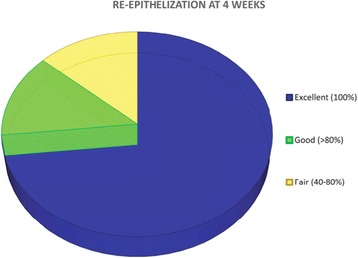
Re-epithelization at 4 weeks

A total of 15 patients were enrolled for the study, 12 females and 3 males, aged between 43 and 72 years old. Of these patients, 11 received surgery in the mandible and 4 in the maxilla. No complications were registered during surgeries and the immediate post-operative course was uneventful for all patients. At 1 year, 2 patients dropped out of the study: the first patient experienced a peri-implantitis that was solved with a conventional free gingival graft procedure while the second one developed peri-implant pockets with perimucositis, making the 1-year analysis possible only on 13 patients. No allergic reactions were registered during the clinical trial. The average keratinized tissue width before surgery was 0.4 mm. The initial gain in keratinized tissue (as measured immediately post-op) was 5.7 mm. Constant contraction of the grafted area, and decrease in keratinized tissue width, could be observed throughout 5 years of follow-up (Fig. 2). The initial 6 months were the most critical, with the highest reduction, resulting in a mean of 2.2 mm loss (37%) of value. Another 0.5 mm were lost in the following 6 months (leading to the 1 year examination time point), raising the value of contraction to 48%. After 1 year, the contraction considerably slowed down, with a further 11% width loss over the subsequent 4 years. The total loss after 5 years of follow-up was a mean value of 3.3 mm, corresponding to 59% of the initial measurement (Fig. 3). Clinically, it was assessed that the height of the keratinized tissue was, in any case, lower than the adjacent sites. Re-epithelization was excellent and complete in most patients after 4 weeks (Fig. 4). The matrix also proved to be a great hemostatic, with no bleeding referred by subjects in the post-op period. The procedure was short, with a mean operative time of 20 min from first incision to last suture, and painless, as referred by patients to the examiners during the first two follow-ups (Mankoski Pain Scale value of 1–2). No visible difference in texture and color could be found, after healing was complete, between the grafted area and surrounding tissue, ensuring a great esthetic outcome over the entire 5 years of follow-up.

## Discussion

The study was carried out to evaluate the efficacy of a xenogeneic CM when used as a soft tissue substitute in the reconstruction of an adequate amount (at least 2 mm) of keratinized tissue around dental implants. The xenogeneic CMs have already been investigated in order to check their compatibility and effectiveness as scaffold [19, 20]. One of the first studies was conducted by Schoo and Coppes, who experimented the capacities of a freeze-dried dura mater grafting material in stimulating keratinized mucosa, with very poor results [21]. Two studies by Harris, in 2001, analyzed the usefulness of acellular dermal matrices when positioned upon the periosteum and bone [22, 23], without any particular results. Furthermore, in 2001, Wei P. and Laurell L. conducted two studies: one clinical and one histological [24]. Six patients received an autologous graft while the remaining six an allogeneic one. The increment in adherent mucosa was detectable in both groups, but the gain was very little with the allogeneic graft. This was related to an excessive shrinkage of the graft post-operatively. Histologically, it was observed that all grafted sites showed a scar-like tissue, incapable of inducing cellular differentiation. The fast resorption represented a big concern in this technique leading to a disturbed healing and sequentially to a lack of keratinized tissue. Harris et al. underlined the great esthetic outcome in addition to the efficacy of substitutive matrices for soft tissue [25]. Despite the physical and mechanical characteristics of these matrices are still under investigation, it has already been observed how these matrices show statistically significant results confronted with the autologous grafting techniques [26]. Comparable results were observed evaluating not only by keratinized mucosa and thickness and vestibular depth evaluation but also by histological study [27]: during the first week of healing, it has been noticed a tissue remodeling due to phagocytosis of pre-existing collagen fibers by macrophages. After 2 weeks, new collagen fibers were detected as long as neoangiogenesis and re-epithelization on the membrane surface. At 4 weeks, it was difficult to find pre-existing collagen fibers. At 10 weeks, the healing process was complete and the esthetic is already acceptable. Schmitt et al. achieved similar outcomes in their study, which compared free gingival grafts and a porcine CM [16]. At 90 days after surgery, biopsies were harvested for histologic and immunohistologic analyses. It was observed the presence of specific keratinized tissue markers is in the CM grafted areas. CMs were also clinically tested as an alternative option for root coverage. McGuire and Scheyer tested the CM associated to a coronally advanced flap in recession defects [28]. They find out how the porcine CM in combination with a coronal flap represented a satisfactory alternative to autografts for covering dehiscence-type recession defects. They also noticed a reduction of the morbidity due to the absence of soft tissue graft harvesting. Nevins M. et al. tested the porcine CM around a single tooth [11]. After 1 year from surgery, both xenogeneic and autologous grafts healed perfectly, showing mature connective tissue and the presence of enough keratinized mucosa. The author underlined the better esthetic outcome of the CM, which was showing a perfect tissue integration. Herford et al. investigated the efficacy of CMs for keratinized mucosa augmentation in cases of lack [29]. They demonstrated an overall mean shrinkage of 14% (range, 5 to 20%). Sanz et al. confirmed how xenogeneic CMs guarantee predictable and satisfactory results: their primary endpoint was to evaluate the potentiality in gaining keratinized tissue in comparison with an autologous graft [16]. At 6 months, they observed an insignificant statistical variance between the autologous (60% shrinkage) and the xenogeneic (67%). Despite the CM shrinkage range is still very wide (from 14 to 75%), most of the authors observed that the majority of shrinkage occurred in the first month after surgery. In the present study, the highest shrinkage rate was observed in the first 6 months (*P* = 0.002) while the following follow-up at 1, 4, and 5 years. The shrinkage was slower but reached a final value of 59%, which is included in the range observed in the other studies. In the present paper, by analyzing the post-operative course, one factor is particularly highlighted: the great decrease in morbidity. Post-operative pain in autologous grafting is caused by the presence of a second surgical site, the donor site. Most of the patients who underwent this type surgery did not feel any pain at all, except a little nuisance that required a mild analgesic as medication. In the present study, the grafts were additionally covered with a vestibular retention splint. The use of the vestibular retention splint guaranteed a mechanic protection for the grafts. Moreover, it had a preventive effect on reinsertion of vestibular muscle fibers. Despite the influence of the splint on the width of keratinized mucosa has not be taken in consideration in this study, Heberer et al. previously concluded its use after vestibuloplasty reduced the graft shrinkage [30]. Furthermore, they observed a general reduction of time of the surgery and post-op morbidity too. The CM is extremely easy to use, with an average length of surgery of 30 min, excluding anesthesia [16]. Intra-operative and post-operative bleeding was extremely limited. The xenogeneic CM showed an ideal haemostatic effect with no excessive bleeding during surgery and no bleeding at all during post-operative period, similar to other techniques using absorbable collagen sponges in different kinds of treatments [31]. Furthermore, all grafted sites presented, when healing was completed, an optimal integration with the surrounding tissues, as stated by other authors in previous studies [15–17]. Rotundo an Pini-Prato observed the good esthetic of the CM also when used in the coverage of multiple gingival recession [32]. Laino et al. described excellent results of CM in wound repairing when placed after intraoral mucosal biopsy [33]. Recently, Schmitt et al. observed the long-term efficacy of CM when used in vestibuloplasty when compared to free gingival graft [34]. Despite the free gingival group showed lower values of keratinized tissue resorption, the CM showed good stability and better esthetic outcomes. Further studies with a larger sample, investigating the long-term effectiveness of CMs and alternative treatment options, should be performed in the future to better assess a univocal outcome about the topic.

## Conclusions

With the limits of this study, it can be assessed that the CM is an effective option for the keratinized tissue augmentation. The percentage of shrinkage of the graft is comparable to data recovered from other studies and does not represent a problem also after 5 years. The CM integration is slow and constant, providing the necessary scaffold to regenerate keratinized mucosa and ensuring a perfect healing. Patients reported no bleeding, and post-operative morbidity was very low. Observing the grafted areas, it is possible to notice the high esthetic result without any dyschromia with the surrounding tissue. This study shows that this type of CM can find major interest in those patients who need a keratinized tissue augmentation around implants with great esthetic outcome or in those who can bear little pain. Further studies will be necessary to assess definitively the efficacy and the applications of this material, eventually gaining statistical value.
